# National health policy-makers’ views on the clarity and utility of *Countdown to 2015* country profiles and reports: findings from two exploratory qualitative studies

**DOI:** 10.1186/1478-4505-12-40

**Published:** 2014-08-15

**Authors:** Benjamin M Hunter, Jennifer H Requejo, Ian Pope, Bernadette Daelmans, Susan F Murray

**Affiliations:** 1 King’s College London, International Development Institute, Chesham Building, The Strand, London WC2R 2LS, UK; 2 Johns Hopkins University, Johns Hopkins Bloomberg School of Public Health, N Wolfe Street #5041, Baltimore, MD 21205, USA; 3 Partnership for Maternal, Newborn and Child Health, World Health Organization, Avenue Appia, CH 1211 Geneva 27, Switzerland; 4 Bart’s Health NHS Trust, W Smithfield, London E13 8SL, UK; 5 World Health Organization, Department of Maternal, Newborn, Child and Adolescent Health, World Health Organization, Avenue Appia, CH 1211 Geneva 27, Switzerland

**Keywords:** Developing countries, Exploratory research, Health policy, Health services research, Policy-making

## Abstract

**Background:**

The use of sets of indicators to assess progress has become commonplace in the global health arena. Exploratory research has suggested that indicators used for global monitoring purposes can play a role in national policy-making, however, the mechanisms through which this occurs are poorly understood. This article reports findings from two qualitative studies that aimed to explore national policy-makers’ interpretation and use of indicators from country profiles and reports developed by Countdown to 2015.

**Methods:**

An initial study aimed at exploring comprehension of Countdown data was conducted at the 2010 joint Women Deliver/Countdown conference. A second study was conducted at the 64^th^ World Health Assembly in 2011, specifically targeting national policy-makers. Semi-structured interviews were carried out with 29 and 22 participants, respectively, at each event. Participants were asked about their understanding of specific graphs and indicators used or proposed for use in Countdown country profiles, and their perception of how such data can inform national policy-making. Responses were categorised using a framework analysis.

**Results:**

Respondents in both studies acknowledged the importance of the profiles for tracking progress on key health indicators in and across countries, noting that they could be used to highlight changes in coverage, possible directions for future policy, for lobbying finance ministers to increase resources for health, and to stimulate competition between neighbouring or socioeconomically similar countries. However, some respondents raised questions about discrepancies between global estimates and data produced by national governments, and some struggled to understand the profile graphs shown in the absence of explanatory text. Some respondents reported that use of Countdown data in national policy-making was constrained by limited awareness of the initiative, insufficient detail in the country profiles to inform policy, and the absence of indicators felt to be more appropriate to their own country contexts.

**Conclusions:**

The two studies emphasise the need for country consultations to ensure that national policy-makers understand how to interpret and use tools like the Countdown profile for planning purposes. They make clear the value of qualitative research for refining tools used to promote accountability, and the need for country level Countdown-like processes.

## Background

The development of national health policies is a complex process that remains poorly understood [1-5]. Commentators have highlighted the role of national and regional political processes [6,7], power structures [8], and, increasingly, global governance and the global health arena in policy-making [9,10]. Qualitative studies have provided the greatest insight into the influence of the latter on national policy-makers, suggesting that international meetings and networks contribute to the formation of ‘norms’ for national health policies [9-11], and that funds and technical assistance from international donors guide the development and implementation of such policies [9,10,12,13]. Research has suggested that ‘evidence’, when presented clearly and concisely, can play an important role in policy-making [14-16]. Less attention has been devoted specifically to the impact of tools used for global monitoring, for example country profiles, on national policy-making. Here, we report on two exploratory studies that investigated this area.

Monitoring and accountability systems of donor governments and international agencies often include a core set of health and development indicators that are tracked over time to assess progress [17-22]. The small existing literature suggests a somewhat mixed experience with the usage of such indicators among national policy-makers in low- and middle-income countries. Exploratory research has indicated that data used for global monitoring can influence national policy-making processes [23], particularly when evidence is presented in high impact international journals or by United Nations agencies [24,25]. However, complex reporting requirements from donor institutions and governments have been shown to create a burden on already over-stretched local and national governments [26].

Critiques of global monitoring efforts suggest that the collection of a limited number of quantitative indicators runs the risk of focusing attention on meeting a set of narrow targets rather than achievement of equitable gains in health and the broader aspirational goal of development [27,28]. The use of data from the global health arena to compare countries’ health system ‘performance’ has perhaps generated the most controversy, in particular the ranking of national health system performance used in the World Health Organization’s (WHO) *World Health Report, 2000*[29]. Concerns were voiced that the methodology for generating the measures and rankings was difficult to understand, and that national policy-makers would be embarrassed by poor results relative to neighbouring countries [30,31]. There have been varied views as to whether the resulting debate improved methods for assessing health system performance [30,32], or detracted from key messages in the report for improving national health systems [33].

Our article contributes to this literature with an account of qualitative research on national policy-makers’ and their partners’ perceptions of data presented in country profiles and reports prepared by Countdown to 2015 for Maternal, Newborn and Child Survival (Countdown). Countdown is a global initiative that reports on select indicators in 75 ‘high-burden’ countries to stimulate attention and financial resources towards achieving the health-related Millennium Development Goals [18,34]. The initiative focuses on coverage of proven maternal, newborn, and child heath interventions and key determinants of coverage such as equity patterns [35,36], policy and health system measures, and data on financial flows to women’s and children’s health [37]. Information on the country selection process and indicators tracked by Countdown is available at http://www.countdown2015mnch.org.

The two exploratory studies described in this article examined perceptions of country profiles from the *Countdown to 2015 Decade Report (2000–2010): Taking stock of maternal, newborn and child survival*[38]. The aim of the first study was to assess whether a subset of charts on, or proposed for inclusion in, the country profiles were effective in communicating the intended information to Countdown’s target audiences. The second study explored national policy-makers’ understanding of selected Countdown indicators and their perceptions regarding the utility of such data.

## Methods

Countdown conducts a rigorous technical review process of the indicators it tracks approximately every two years to ensure that these indicators reflect current evidence on the continuum of care for women’s and children’s health. As part of this process, two independent studies using qualitative methods [39,40], each supported and approved by Countdown, were conducted to explore perceptions of the indicators and graphs on the Countdown country profiles. Countdown reports were only available in English when the two studies were conducted.

The first study was conducted in 2010 at a joint Women Deliver/Countdown conference in Washington DC, where the *Countdown to 2015 Decade Report (2000–2010)* was launched. The first study had two objectives: 1) to examine the extent to which policy-makers and partner organisations (such as academics, donor organisations, and international and national non-governmental organisations) understood the information in the graphical representations of water and sanitation coverage, infant and young child feeding practices, and inequities in coverage, and 2) to examine how the graphs on the profiles could be improved to more effectively convey information to Countdown’s intended audiences. Ethical approval for the study was obtained from Johns Hopkins University. Details of the methods used are described below and replicas of the graphs that were used are available in Additional file 1. The full study is available upon request from the authors.

### Methods used in study one

Respondents were participants at a joint Women Deliver/Countdown conference held in Washington DC who were selected through two recruitment strategies. The first strategy involved identification of policy-makers from Countdown countries and their partner organisations from the conference registration lists. These potential respondents were contacted via email prior to the conference with an invitation to participate in the study and a time and location for the interview was agreed upon. The second strategy involved on-site recruitment through a personal request from Countdown members during the conference.

A total of 27 interviews with 29 respondents were completed. Special efforts were made to obtain a sample that was evenly divided between policy-makers from Countdown countries and representatives of partner agencies. Almost two-thirds of respondents were female, and over twice as many respondents were from North America than from any other region. Over half of the respondents worked in development partner agencies (15), others included ministry of health representatives (3), professors (6), clinicians (2), graduate students (2), and other (1). Participants were approached by Countdown members who explained the purpose of the study, and verbal consent in English was obtained from each respondent. The interviews were conducted at a location in the conference centre and at a time during the conference considered convenient by the respondent, and took approximately 30 minutes to complete.

Interview questions were developed by the study team and were directly related to the study objectives. Each respondent was shown four charts and asked four semi-structured questions: what information they thought each chart displayed, how they interpreted the information, whether they thought it matched Countdown’s intended message, and what (if any) changes they would make. The four charts, which were used in the country profiles or proposed for use by Countdown, had been selected by the Countdown Coordinating Committee based on their perception that they might not be readily understood by the target audiences due to their complexity. These included graphs on infant and young child feeding, equity, and water and sanitation.

All interviews were conducted in English by the same interviewer, although one respondent was accompanied by a translator. Each interview was audio recorded and transcribed using Microsoft Word. All members of the Women Deliver study team independently read the interview transcripts and, using a consensus process, organised the question responses into the following thematic areas that responded to the main aims of the study: 1) consistency between the respondent’s interpretation of the chart and the message intended by Countdown; 2) perception on whether the chart communicated the intended message; 3) suggestions on how to improve the information presented in the chart, including the chart’s format and layout.

The second study was conducted during the 64^th^ World Health Assembly in Geneva in 2011. There were five objectives: 1) to generate feedback on the clarity of data presentation in the *Countdown to 2015 Decade Report (2000–2010)*; 2) to highlight any additional data that could be included in Countdown country profiles; 3) to explore the views of national policy-makers on Countdown and its processes; 4) to examine their perceptions and experiences of the usefulness of health indicators for guiding policy; and 5) to determine national policy-makers’ views on health data that compare countries. Ethical approval for the study was obtained from WHO and King’s College London. Details of the methods used are described below. The full study is available upon request from the authors.

### Methods used in study two

Recruitment of interviewees and data collection took place during the 64^th^ World Health Assembly in Geneva. Sampling was purposive and aimed to obtain a range of national policy-makers on the following dimensions: a cross section of WHO regions, a variety of levels of previous engagement with the Countdown process and a range of policy-making responsibilities. One of the authors (IP) approached delegations from low- and middle-income countries to explain the study. Delegations that agreed to participate nominated one or two team members who they felt was most suitable to take part on their behalf. Twenty interviews were conducted in English, French, or Spanish with 22 senior policy-makers from South Asia (1), East Asia/Pacific (3), Eastern/Southern Africa (9), West/Central Africa (3), Middle East/North Africa (2), and Latin America/Caribbean (2). Half of the countries sampled for the study had been ‘rapid returners’ of a questionnaire that collected data for the Countdown decade report, the other half were not. The interviewees included a Vice Minister of Health, Directors of Maternal and Child health, Director-Generals of Health Services, and Senior Technical Advisors to Ministries of Health.

Interviews were conducted immediately after approaching the delegation, or at a later time that was more convenient for the participant. The aims and methods of the research were explained to each participant and an English language information sheet was provided. Interpreters explained the research to participants who preferred to communicate in French or Spanish and in these cases the interpreter then translated and read the information sheet to the participant. Delegates who agreed to participate in the study signed an English language consent form to indicate that they understood the purpose of the research and agreed to the publication of anonymised data.

Interviews were conducted at a time and place suggested by each participant and ranged from 10 to 45 minutes in length. Participants were shown the *Countdown to 2015 Decade Report (2000–2010)* and a semi-structured interview guide was used to ask questions relating to each of the five study objectives. Examples included if they had heard about the Countdown initiative, how they felt about comparing data from different countries, and their understanding and perceived usefulness of three indicators from the *Countdown to 2015 Decade Report (2000–2010)*, namely community treatment of pneumonia with antibiotics, specific notification of maternal deaths, and out-of-pocket expenditure as a proportion of total expenditure on health. Similar to the first study, these three indicators had been selected by the Countdown Coordinating Committee on the basis that they might not be readily understood by target audiences.

Interviews conducted in English were transcribed verbatim by IP using Microsoft Word. For interviews conducted in French or Spanish, simultaneous translation into English and transcription was conducted by a bilingual interviewer and researcher (IP), allowing for clarification of expressions and meanings to occur. All but one of the interviews were audio recorded. In the case where recording was not permitted by the interviewee, IP took detailed notes. Data were analysed using a framework analysis approach [41]. Transcripts were read closely by one of the co-authors (IP), who identified three major thematic areas relating to the aims and objectives of that study: perceived influence of international initiatives on policy-making, opinions on data that compare countries, and perceptions of Countdown’s data. Transcripts were indexed and the data then arranged in thematic charts and analysed within each of these. Their fit was then verified by another co-author (SM).

The two studies explored overlapping issues and their results were complementary. Here, we adopt a convergent approach for data synthesis [42] and organise results into three overarching themes that related to participants’ comprehension of the information presented in the Countdown country profiles, their perceptions of the value of the profiles for guiding decision-making and their mechanisms of influence, and their views on data that compare countries.

## Results

### Study one

#### Comprehension of Countdown data

Respondents at the Women Deliver/Countdown conference agreed that the topic areas represented in each of the four charts were essential for understanding a country’s progress in improving maternal, neonatal, and child health (improved water sources shown as a stacked bar chart, improved sanitation facilities shown as a stacked bar chart, infant and young child feeding by age presented as an area chart, and equity graphs displaying the coverage gap and floating bars to show how coverage ranges by wealth quintile). However, approximately one-third of respondents from the Women Deliver/Countdown conference reported that they did not understand the intended meaning of the charts when viewed without supporting documentation and out of context of the full two-page profile. Almost all participants made suggestions on how each chart could be improved. Although comments on specific graphs varied, several general views were expressed:

•Most respondents indicated that the intended meaning of the charts was not immediately evident upon visual inspection alone.

•Area charts (line charts that include filled-in colour below the line to indicate volume), such as the proposed infant and young child feeding area chart, were particularly challenging for respondents to interpret without accompanying text given their complexity. Most respondents, for example, felt that the mixed feeding categories on the chart did not provide enough programme relevant information on what proportion of a child’s diet was attributable to each feeding category. They also noted that it was difficult to interpret the values on the chart without reference points or evidence-based benchmarks for what each country should be striving to achieve per infant feeding category shown.

•Respondents assumed that the labels and text in the chart would further clarify what information is being conveyed, and chart titles were viewed first for an explanation on the graph contents. Respondents reported that, for some charts, the labels and text conveyed something different from the title or from their interpretation of what was shown in the graph. Respondents noted, for example, that the equity floating bar chart subtitle (‘mean coverage levels for country X in the poorest and richest quintiles for selected interventions along the continuum of care’) was inconsistent with the floating bars presented in the graph which showed coverage across all quintiles. For the water and sanitation charts, respondents recommended revising the title to include the time frame of the data shown, namely 1990–2008.

#### Value of the report and mechanisms of influence

In general, participants from the Women Deliver/Countdown conference felt that to maximise the impact of the country profile and its value for use as a decision-making tool, all graphs should make a strong statement with clear implications for health policy formulation. The general consensus from respondents was for Countdown to consider developing brief paragraphs on how to interpret the profile charts so that the policy implications are evident, and to include these in the report annexes or alongside the profiles.

Recommendations from the first study were presented upon completion to the Countdown Coordinating Committee as part of the initiative’s technical review process for developing the 2012 country profiles. These recommendations were considered as key inputs to the process, which involved a review of new epidemiological evidence on effective interventions for reproductive, maternal, neonatal and child health, data availability for each indicator tracked across Countdown’s 75 priority countries, and assessment of the format of the two-page profile template. A set of decisions were reached on changing the profile template, indicator list, and presentation of specific charts (see Additional files 2 and 3 as examples of profiles in the 2010 report and the 2012 report, respectively).

### Study two

The second study focused on national policy-makers at the World Health Assembly and findings were organised by three themes: participants’ comprehension of the information presented in the Countdown country profiles, their perceptions of the value of the profiles for guiding decision-making and their mechanisms of influence, and their views on data that compare countries.

#### Comprehension of Countdown data

Most participants from the World Health Assembly felt that the specific health systems and policy indicators they were asked to interpret (community treatment of pneumonia with antibiotics, specific notification of maternal deaths, and out-of-pocket expenditure as a proportion of total expenditure on health) were important. However, levels of comprehension varied and some interviewees were unsure how to interpret some of the indicators or the graphics used to present them in the Countdown report.

“*I never know how you calculate them myself. Like out-of-pocket expenditure as percentage of total expenditure on health – since I don’t know how it's calculated it doesn’t tell much.*”

Although the specific indicators tracked by Countdown were generally felt to be a useful indication of situations and trends, some participants suggested that they lacked enough detail to inform their policy-making. Others noted that the subset of indicators tracked by Countdown may not include indicators felt to be more important in specific countries:

“*If what we are looking for is a general feeling of whether there is some access to treatment generally, then yes this is ok, but if what you are looking for is whether the children are being appropriately managed, this doesn’t tell you.*”

“*I don’t find* [specific notification of maternal deaths] *useful, this is not very specific … the way it has been framed, because specific notification of maternal deaths, you know the indictors itself, is not specific.*”

“*This one on out-of-pocket expenditure is not interesting. So before, yes, we used this indicator but because we moved to an insurance model with universal coverage model, so this is no longer interesting.*”

#### Value of the report and mechanisms of influence

Interviews at the World Health Assembly revealed a lack of awareness of Countdown:

“*Personally, it’s the first time that I have seen this document… but I think in the future by reading the document I think if the information is divulged and shared by decision makers I think that that can effectively influence the decision to be taken.*”

More than a third of participants recruited at the World Health Assembly said they were unaware of Countdown prior to the approach for the interview. Of those who had not heard of Countdown, the majority were French or Spanish language speakers.

Participants from the World Health Assembly who were aware of Countdown highlighted several mechanisms through which Countdown data influences policy-making processes. Some participants felt the indicators helped to illustrate changes in coverage in their country and possible directions for future policy:

“*It’s very useful, in that it helps us take stock, so it is very useful for us, because we can see exactly where we are, regarding the key interventions, the key high impact interventions.*”

“*I see that with child health we have moved quite fast, and look at the key areas of high impact intervention in terms of coverage, when I am looking at the page here I can quickly tell that we need to do something on pneumonia, we need to do something on nutrition. Those are critical. Immunisation, I can safely say that we are where we are supposed to be but we need to sustain that so we don’t let it drop.*”

Other participants at the World Health Assembly described how they have been able to use indicators such as those presented in Countdown reports to lobby other stakeholders and departments in their national government to increase resources for health:

“*Now* [the President] *is talking more about maternal and child health because of this kind of information. The provincial governors are talking more about water and sanitation and* [they have provided] *orientation to all government members that they have to talk about some specific issues according to the evidence we have.*”

“*It is helpful especially to educate our political leaders so they are able to put their resources where* [they] *matter most. We bureaucrats, we can make good policy but if we don’t have good support in resource allocation we won’t get anything.*”

#### Views on comparing progress across countries

Participants from the World Health Assembly expressed a mixed reaction to the use of standardised indicators to compare progress across countries, though few were opposed to the idea. Some felt that unfavourable comparisons with neighbouring countries provided an impetus to improve, and that lessons could be learned from successes and challenges experienced in other countries:

“*Yeah* [Countdown] *has influenced policy because we sort of like refer to it when looking at the majority of these influences here and seeing how we compare with sister countries and nobody wants to be the worst performer.*”

“*It is helpful, because for me I have never encountered other sub-Saharan countries so if I see someone has done well I look at the lessons learnt and also the constraints or challenges. So there is cross-pollination of ideas, and also it assists you not to do the same thing which is not successful. So I find it useful, I use it quite a lot.*”

Others were more sceptical of comparing progress across countries, noting that data in the Countdown country profiles differs from data on the same indicators released by some national governments, and pointed out that such comparisons risked offending policy-makers in some countries:

“*These same data and indicators are collected in different ways depending upon the partners who are assisting in the data collection or depending on the systems which are used to collect the data … there is a lot of discussion and discordance that is happening for data, especially those related to maternal mortality, as the data from countries are different from the data that partners are publishing.*”

“*There is some sensitivity in ranking. It has to be* [done] *with caution but is useful because many countries that find themselves below certain acceptable ranking for themselves particularly feel that they need to do something. So it is useful, but we also know the reaction in 2000 when the WHO did the health systems ranking, that there was unhappiness and maybe the unhappiness was because there was a sense from some countries that they weren’t too comfortable with the information that was used.*”

“*You see I don’t know where* [the maternal mortality ratio used by Countdown] *came from. I am in charge of the department … our maternal mortality should not be very high, we have a very high literacy rate, we have a very high family planning indicators, so who is dying? If 65% of our women are using family planning then I go to a country with 12% contraceptive and they have less maternal mortality, to me scientifically and statistically it is wrong.*”

“[Comparisons between countries] *may be important, but it is more that if you know the details not just the figures. They may not help the other country unless you know why this country is like that. Maybe it can just be the beginning point. You need to clarify why is this one doing well and the other is not.*”

## Discussion

The limited awareness of Countdown among some participants at the 2011 World Health Assembly, particularly those from non-Anglophone countries, may have reflected two things. First, the environment of the World Health Assembly, which is less focused on maternal, neonatal, and child health, and the challenges of recruiting participants at that event. Second, poorer penetration of the reports in non-Anglophone countries at that time and the relatively recent translation of Countdown reports into languages other than English.

Countdown is trying to improve its penetration by circulating information periodically through the ‘e-blast’ generated by the Partnership for Maternal, Newborn & Child Health, upgrading its website, increasing the availability of materials translated into French, increasing Countdown representation at key regional and country events throughout the year, including meetings of the Inter-Parliamentary Assembly and joint events held around the UN General Assembly in September, and by packaging the data in new ways such as through short two-four page briefs and other advocacy oriented materials. Countdown has also initiated more country level activities with support from the Bill & Melinda Gates Foundation and the Canadian Department of Foreign Affairs, Trade and Development. These include a portfolio of in-depth case studies to explore how and why countries have made progress towards Millennium Development Goals 4 and 5 using available data on mortality, service coverage, equity, health systems, and policies and finances. Country Countdown processes have been established to stimulate dialogue between key stakeholders about current evidence on women’s and children’s health and to increase Countdown’s visibility and impact at a national level [43,44].

The findings of both studies should be interpreted with caution due to small sample sizes, but they do suggest that, once target audiences are aware of and understand the data presented, the Countdown country profiles and reports can play a role in influencing policy formation and programme implementation in low- and middle-income countries. Findings from interviews during the World Health Assembly suggest that Countdown data can contribute to the areas of the policy process described by Shiffman and Smith as ‘*political entrepreneurship’* and ‘*norm promotion*’ [9,10]. In the area of political entrepreneurship, some policy-makers reported using sets of indicators as a lobbying tool in government to defend and increase the health budget. With regards to norm production, most of the policy-makers in the studies seemed to positively value data that can be used to make cross-country comparisons, noting that this information is helpful for norm promotion, in sparking healthy competition, and for triggering interest in lessons learned from other countries in the same region.

Participants’ responses indicated that some did not understand that indicators by definition serve as signals of where further investigation and action are needed, and are meant to be interpreted in the context of more comprehensive information [45]. Other respondents did not capture the logical flow of the country profile in which mortality data are followed by coverage data of interventions that are critical for mortality, and coverage data are backed up by policy and system indicators, necessary for health system readiness to increase access to and coverage of health services and interventions. Such findings reinforce the need for better channels of communication between researchers and policy-makers, as was highlighted by Innvaer *et al.* in their systematic review of policy-makers’ perceptions of evidence use [14].

The findings of the studies informed Countdown decisions to simplify several of the graphs on the profile and on improving the extent of information provided on the indicators in Countdown reports and annexes. Most of the charts in the 2012 profile are simple bar or line charts, and some titles and sub-titles were revised to be sure they accurately represented the chart contents (e.g., the equity and water and sanitation charts). Key messages were better incorporated into graphs shown in the 2012 Countdown report, with expanded accompanying text to help readers interpret the policy and programmatic significance of the data shown. A traffic-light approach to identifying priority actions, as recommended by participants from the World Health Assembly, was used for presenting the mortality measures in the 2012 report, and the colour scheme of red, yellow, and green is reflected in many of the league tables and bar charts throughout the 2012 report with poorer performing countries shaded in red and higher performing countries shaded in green.

The 2012, 2013, and 2014 Countdown reports include a section on how to use the country profiles, describing each type of indicator on the profile, the range of questions that can be answered by the profile data, how to interpret the values presented for each indicator, and how the information on the profiles can serve as a starting point for further investigation into why progress has or has not occurred. Country-specific slideshow presentations for each of the 75 Countdown countries based on the 2012 profiles that explain how to interpret each profile graph were also prepared and are available on the Countdown website (http://www.countdown2015mnch.org).

Concerns expressed about differences in estimates for specific indicators between national level sources and global databases indicate that more effort is needed to carry-out country consultations on the data prior to the launch of new profiles in order to explain how estimates were derived. This step is critical for country acceptance of the information on the profiles and for their use as a tool in prioritising areas for action and allocation of scarce resources at the national and global level. The Countdown initiative is responding to this call by carrying out in-depth case studies and national Countdown processes that include a review of national data and focus on progress at sub-national level.

Both studies make clear how qualitative studies can help researchers and communication experts alike to better design tools and products like the Countdown profiles in order to reach intended audiences with key messages, and should be an integral and iterative part of the process of developing and disseminating such tools. Such research will have an increasingly important role as part of the post-2015 development framework.

### Study limitations

Both studies adopted a ‘planned opportunism’ approach to interviewing a range of policy-makers and key partners at international meetings. The principal limitation of the studies was in recruiting sufficient numbers of participants from Countdown countries which was, in part, due to the challenges of conducting such research at high level international events. Delegations were extremely busy, for example, in the early days of the World Health Assembly and this reduced the number of interviews that could be conducted for the second study and the interview length since participants had limited time available between meetings.

The restriction of interviews to English only at the Women Deliver/Countdown conference may have limited the representativeness of the participant list. Conducting the interviews in several key languages was important to achieving diversity of participants in the second study, however, this added a layer of complexity to the work. Future exploratory studies could use focus groups or other alternative strategies in addition to interviewing to solicit information about policy-makers’ perceptions of tools like the Countdown profile. Other options for carrying out these studies aside from large, international meetings where there are competing demands on participants’ time should be explored. One such example is online surveys targeted at decision-makers and these were used with some success in a study that examined policy-makers’ perceptions of a framework for evidence-based health policy [4]. Another example is to host an event for policy-makers with the specific purpose of generating and collecting data, as was tried in a study that explored perceptions of using evidence for policy-making among representatives from 11 countries in the Middle-East and North Africa [12].

Despite these caveats, the exploratory qualitative studies reported here are a modest indication of the value of the supra-institutional nature of Countdown, which creates a partnership between academics, other members of civil society, and representatives of UN agencies. In this context, researchers can contribute to the definition and refinement of indicators, as emphasised by Thomas Pogge among others [46], and, through collaboration with communication experts and partners on the ground, improve their use and reporting.

## Conclusions

The two exploratory studies described in this article highlight national policy-makers’ and their partners’ perceptions of data used in Countdown country profiles and reports. Our findings emphasise the need for continued efforts to improve dissemination strategies so that policy-makers in low- and middle-income countries are aware of initiatives like Countdown. They also emphasise the importance of consultation processes with national policy-makers during the preparation and launch of Countdown country profiles and similar tools in order to ensure they understand i) how to use these tools and what the different indicators mean, ii) how to select indicators, iii) how the global databases for indicators such as those tracked in Countdown are compiled and why they may differ from national estimates, and iv) how these data can be used as starting points for further research into understanding data gaps, areas of progress and lack of progress, and for developing supportive policies and programmes.

The findings of these studies provide some insights into the use of global data in national policy-making processes, a step that has been largely overlooked in research to date. The aim of tools like the Countdown country profiles is to support the use of evidence in policy-making, including the identification of priority areas for health systems strengthening. By providing feedback from target audiences, qualitative research, such as the two studies described here, can help initiatives like Countdown refine their tools and reports to better promote the use of evidence for decision-making and accountability at global, national, and sub-national levels. These types of studies should also be undertaken as part of the process of developing goals and targets for the post-2015 global development framework, and communicating their relevance to policy-makers as they, in turn, shape their national level strategies and plans.

## Abbreviation

WHO: World Health Organization.

## Competing interests

The authors declare that they have no competing interests.

## Authors’ contributions

BH and SM conceived the study, and prepared the first draft and revised subsequent versions, JR contributed to the writing of all drafts, and IP and BD reviewed and contributed to the preparation of the drafts. All authors read and approved the final manuscript.

## Supplementary Material

Additional file 1 Figures shown to participants at the joint 2010 Women Deliver/Countdown conference in Washington DC. Click here for file

Additional file 2 **Countdown to 2015 country profile for Bangladesh, 2010.** Source: *Countdown to 2015: Maternal, Newborn & Child Survival.* Click here for file

Additional file 3 **Countdown to 2015 country profile for Ghana, 2012.** Source: *Countdown to 2015: Maternal, Newborn & Child Survival.* Click here for file
