# Supplementary material for: National health policy-makers’ views on the clarity and utility of Countdown to 2015 country profiles and reports: findings from two exploratory qualitative studies
Source: Health Res Policy Syst. 2014 Aug 15;12:40. doi: 10.1186/1478-4505-12-40 (PMC4139135; doi:10.1186/1478-4505-12-40)

**National health policy-makers’ views on the clarity and utility of *Countdown to 2015* country profiles and reports: findings from two exploratory qualitative studies**

**Webannex**

Figures shown to participants at the joint 2010 Women Deliver/Countdown conference in Washington D.C.

Webannex Figure 1.


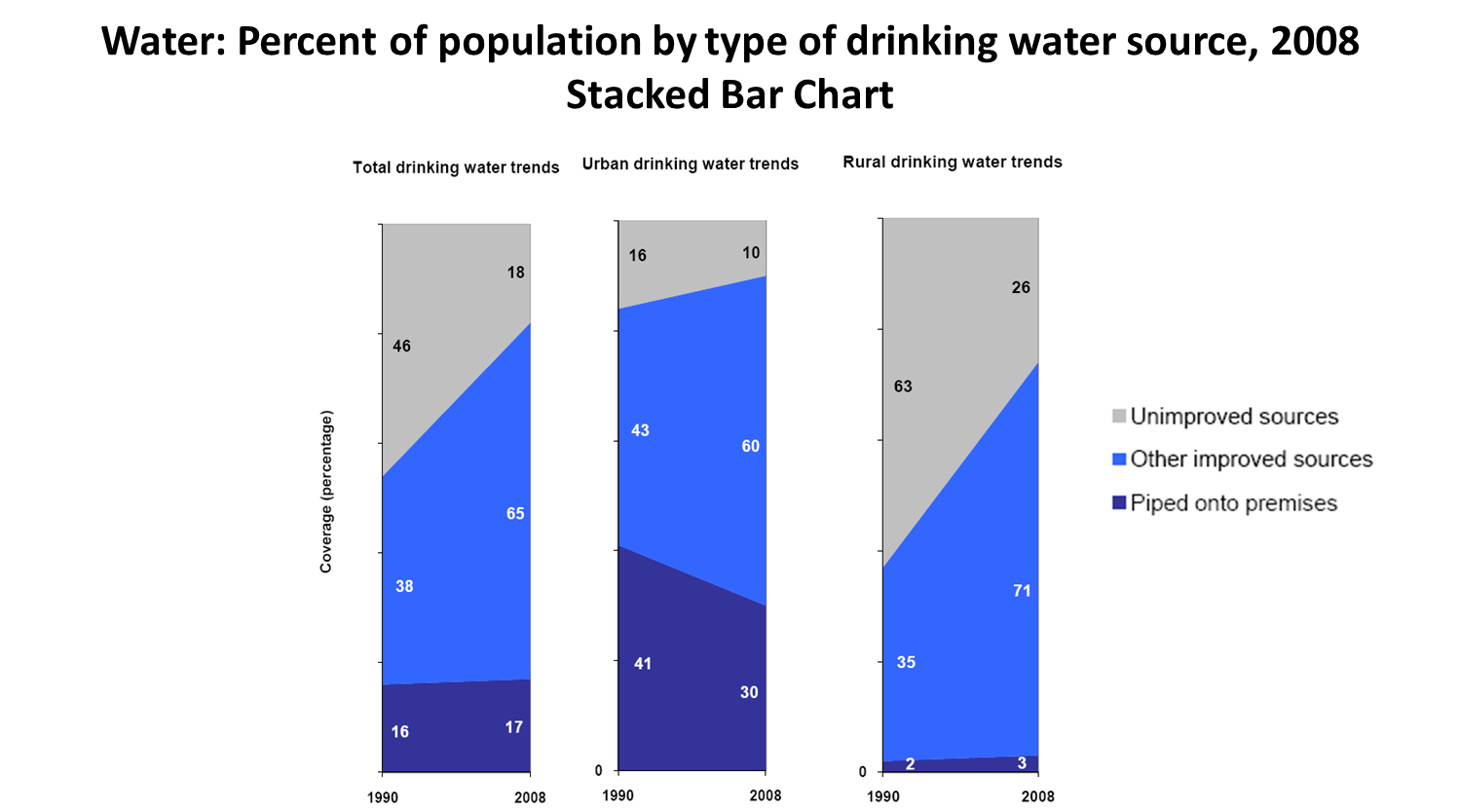


Webannex Figure 2


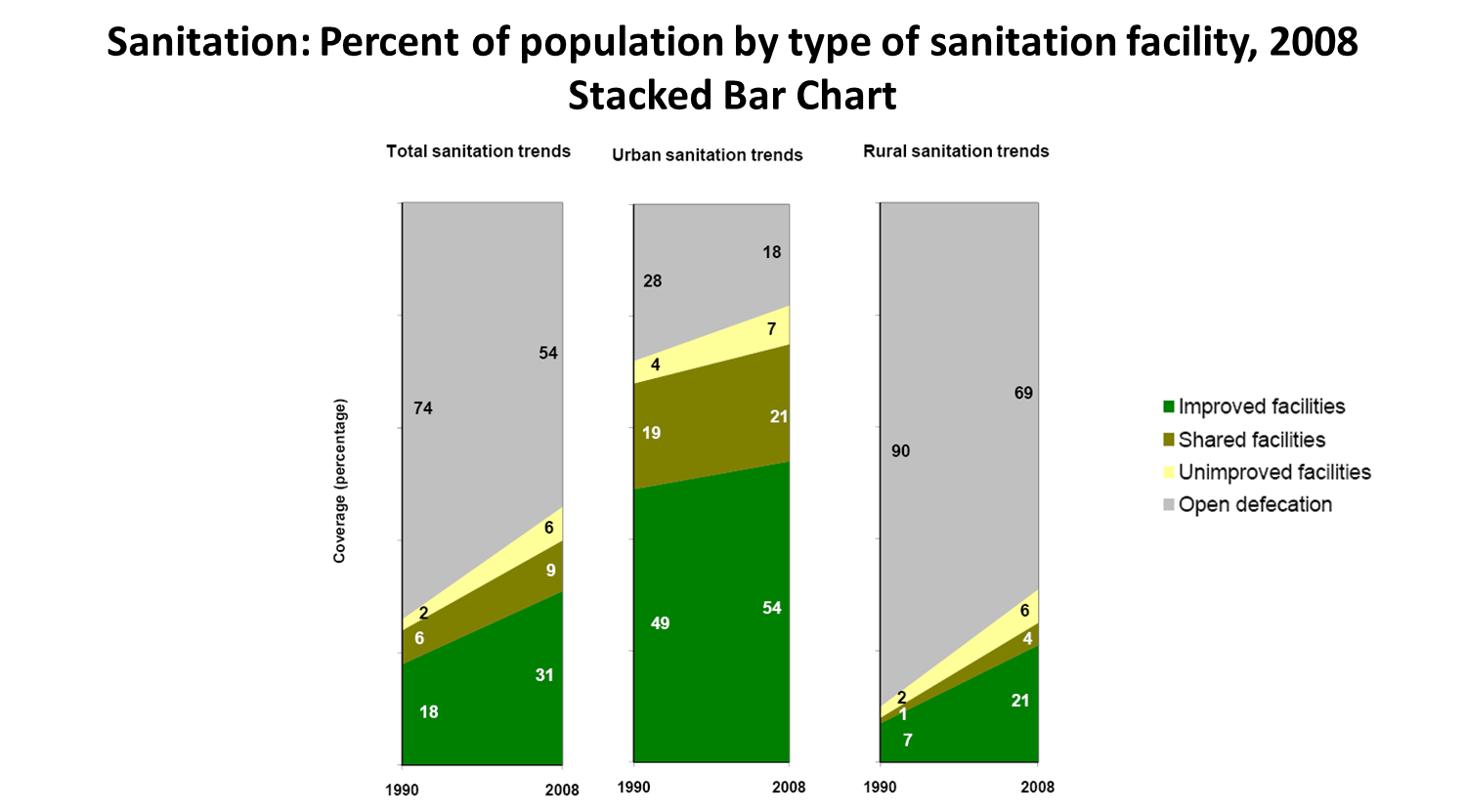


Webannex Figure 3


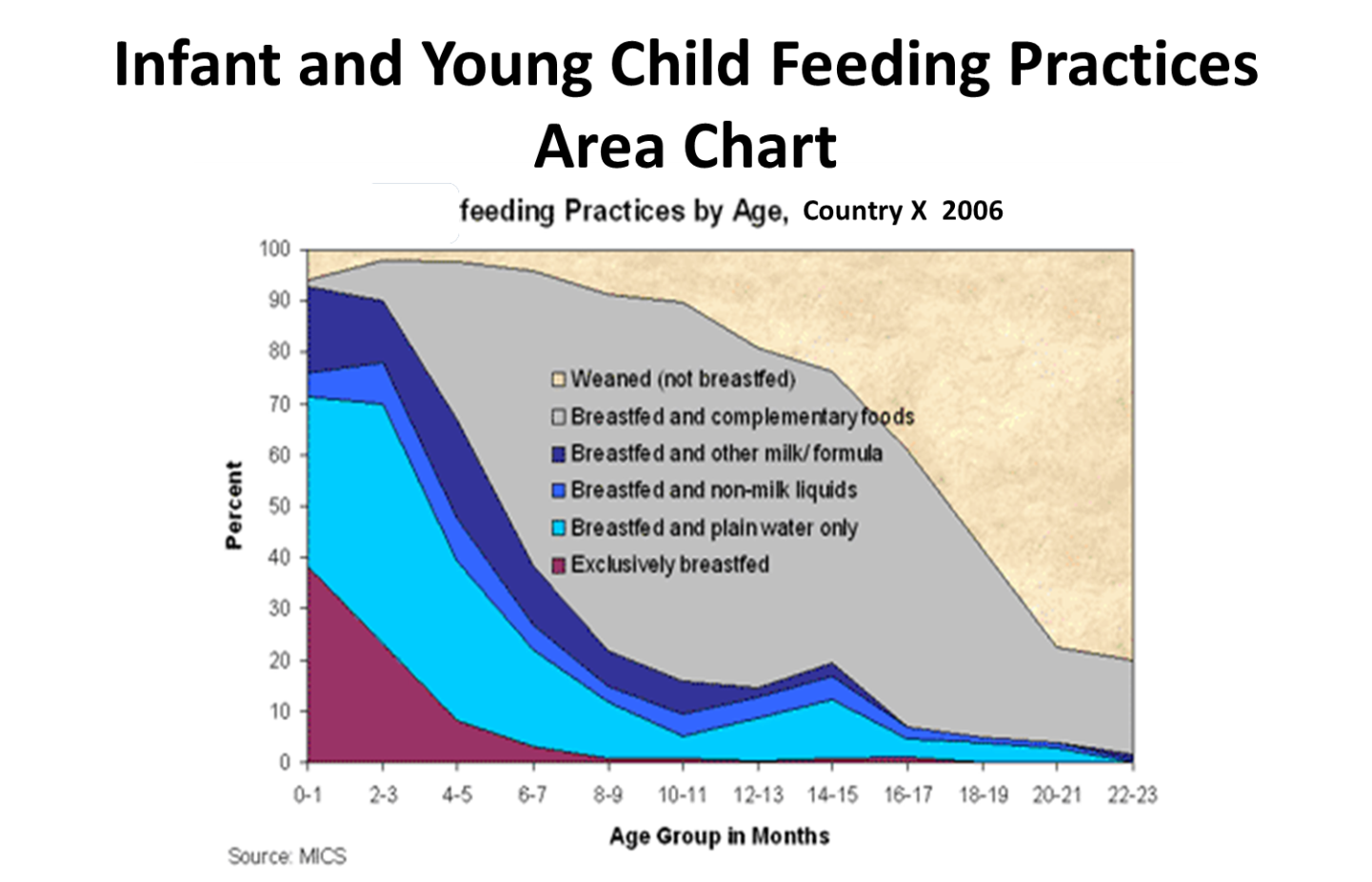


Webannex Figure 4


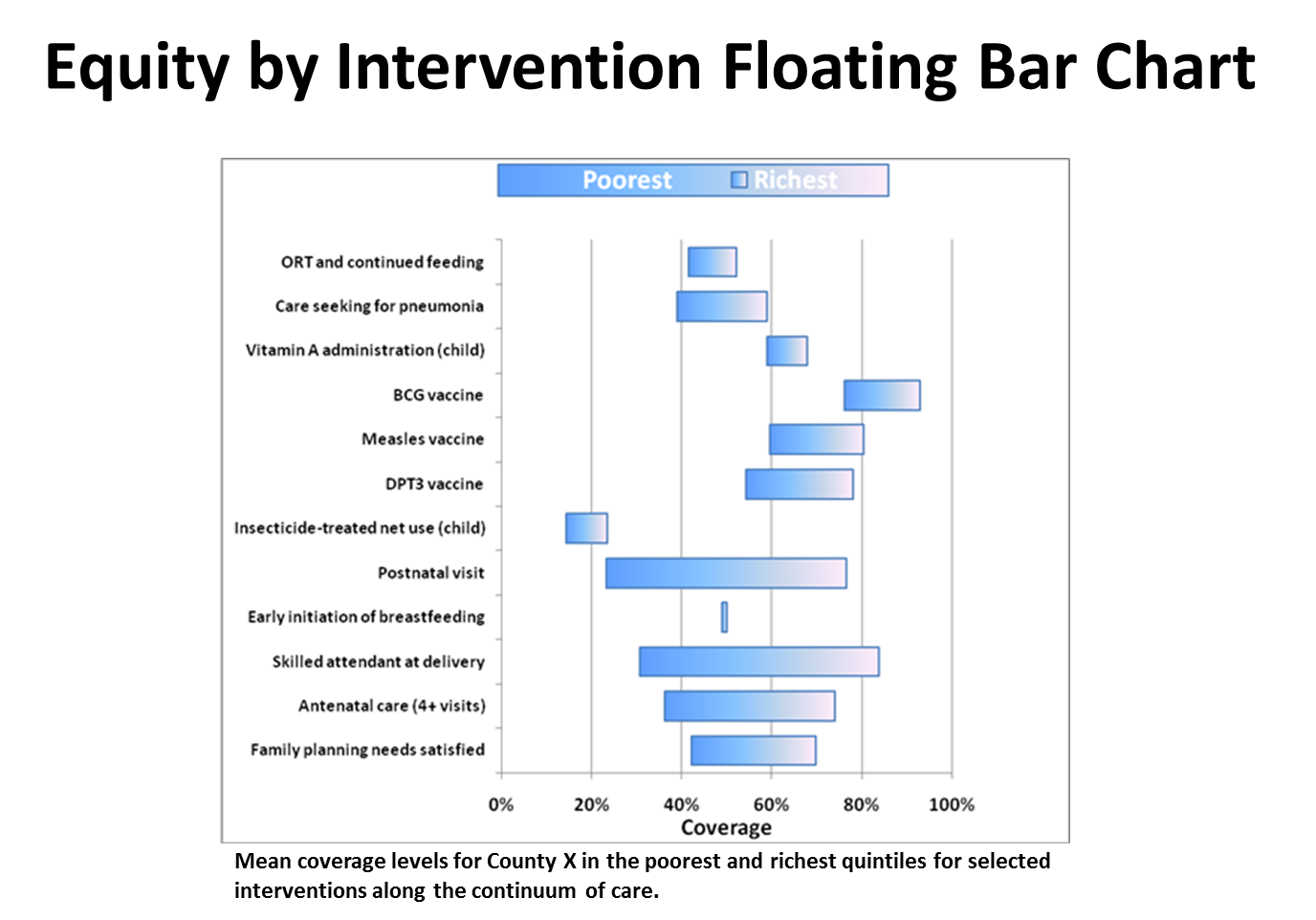


Webannex Figure 5


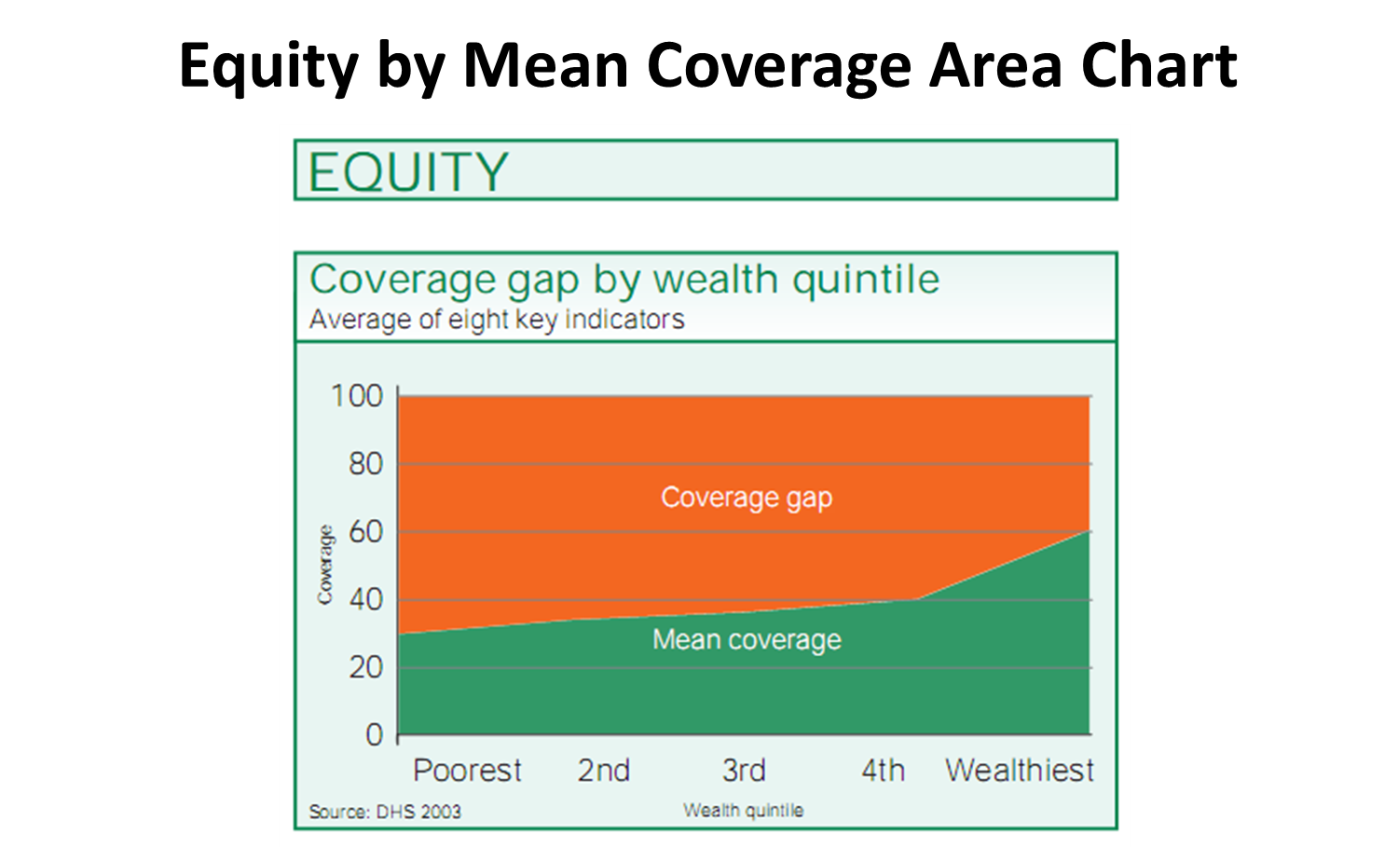

Supplement: Additional file 1 — Figures shown to participants at the joint 2010 Women Deliver/Countdown conference in Washington DC. [file 1478-4505-12-40-S1.docx]
