# Supplementary material for: National health policy-makers’ views on the clarity and utility of Countdown to 2015 country profiles and reports: findings from two exploratory qualitative studies
Source: Health Res Policy Syst. 2014 Aug 15;12:40. doi: 10.1186/1478-4505-12-40 (PMC4139135; doi:10.1186/1478-4505-12-40)
Supplement: Additional file 2 — Countdown to 2015 country profile for Bangladesh, 2010. Source: Countdown to 2015: Maternal, Newborn & Child Survival. [file 1478-4505-12-40-S2.pdf]

Countdown to 2015
Maternal, Newborn & Child Survival

Bangladesh

DEMOGRAPHICS

Total population (000)	160,000	(2008)
Total under-five population (000)	16,710	(2008)
Births (000)	3,430	(2008)
Birth registration (%)	10	(2006)
Under-five mortality rate (per 1000 live births)	54	(2008)
Infant mortality rate (per 1000 live births)	43	(2008)
Neonatal mortality rate (per 1000 live births)	33	(2008)
Total under-five deaths (000)	183	(2008)
Maternal mortality ratio (per 100,000 live births)	570	(2005)
Lifetime risk of maternal death (1 in N)	51	(2005)
Total maternal deaths	21,000	(2005)

Under-five mortality rate
Deaths per 1000 live births

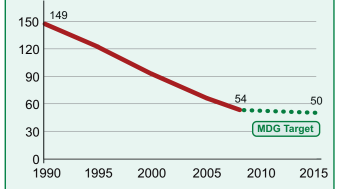

Source: IGME 2009

Causes of under-five deaths, 2008
Globally more than one third of child deaths are attributable to undernutrition

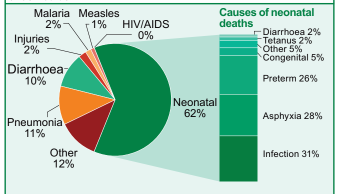

Source: WHO/CHERG 2010 Source: WHO/CHERG 2010

INTERVENTION COVERAGE FOR MOTHERS, NEWBORNS AND CHILDREN

NUTRITION

Stunting prevalence (moderate and severe, %)	43	(2007)	Complementary feeding rate (6-9 months, %)	74	(2007)
Wasting prevalence (moderate and severe, %)	17	(2007)	Low birthweight incidence (%)	22	(2006)

Underweight prevalence
Percent children < 5 years underweight for age*

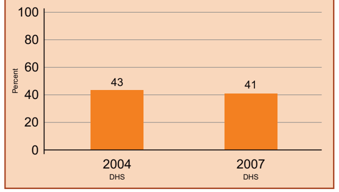

*Based on 2006 WHO reference population

Exclusive breastfeeding
Percent infants < 6 months exclusively breastfed

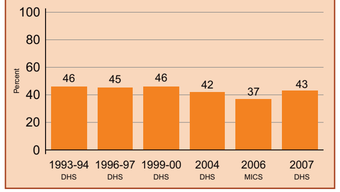

Vitamin A supplementation
Percent children 6-59 months receiving two doses of vitamin A during calender year

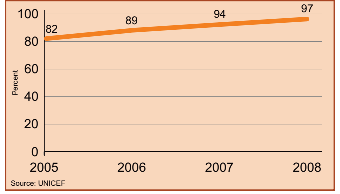

Source: UNICEF

CHILD HEALTH

Immunization
- Percent of children immunised against measles
- Percent of children immunised with 3 doses DPT
- Percent of children immunised with 3 doses Hib

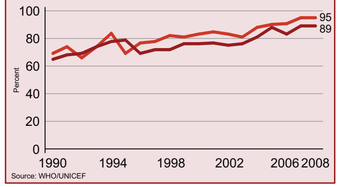

Source: WHO/UNICEF

Malaria prevention
Percent children < 5 years sleeping under ITNs

No data

Prevention of mother to child transmission of HIV
Percent HIV+ pregnant women receiving ARVs for PMTCT

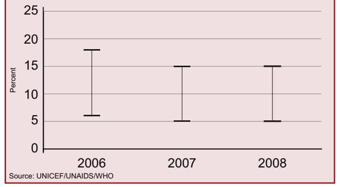

Source: UNICEF/UNAIDS/WHO

Diarrhoeal disease treatment
Percent children < 5 years with diarrhoea receiving oral rehydration therapy or increased fluids, with continued feeding

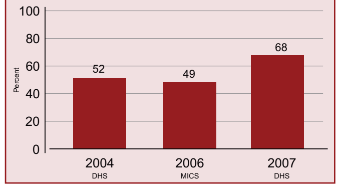

Malaria treatment
Percent febrile children < 5 years using antimalarials

No data

Pneumonia treatment
- Percent children < 5 years with suspected pneumonia taken to appropriate health provider
- Percent children < 5 years with suspected pneumonia receiving antibiotics

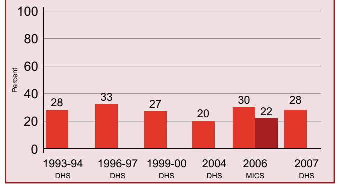

MATERNAL AND NEWBORN HEALTH

Adolescent birth rate (births per 1,000 women)	133	(2005)
Unmet need for family planning (%)	17	(2007)
Antenatal visits for woman (4 or more visits, %)	21	(2007)
Intermittent preventive treatment for malaria (%)	NA*	
C-section rate (total, urban, **rural**; %) (Minimum target is 5% and maximum target is 15%)	8, 16, **5**	(2007)
Early initiation of breastfeeding (within 1 hr of birth, %)	43	(2007)
Postnatal visit for baby (within 2 days for home births, %)	19	(2007)

* Not applicable

Causes of maternal deaths
Regional estimates for South Asia, 1997-2007

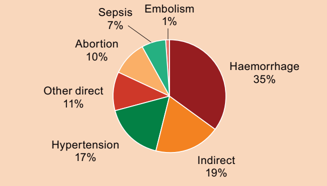

Source: WHO 2010

Coverage along the continuum of care

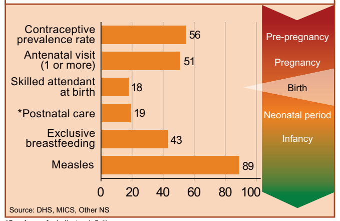

Source: DHS, MICS, Other NS
*See Annex for indicator definition

Antenatal care
Percent women aged 15-49 years attended at least once by a skilled health provider during pregnancy

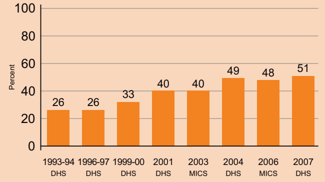

Skilled attendant at delivery
Percent live births attended by skilled health personnel

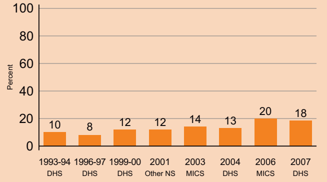

Neonatal tetanus protection
Percent of newborns protected against tetanus

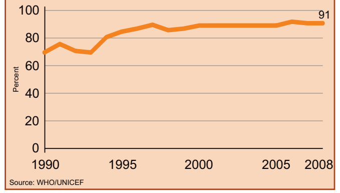

Source: WHO/UNICEF

WATER AND SANITATION

Water
Percent population using improved drinking water sources

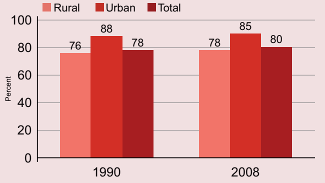

Source: WHO/UNICEF JMP, 2010

Sanitation
Percent population using improved sanitation facilities

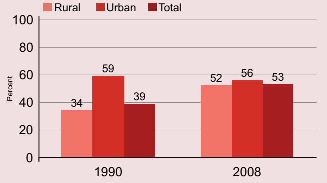

Source: WHO/UNICEF JMP, 2010

EQUITY

Coverage gap by wealth quintile
Average of eight key indicators

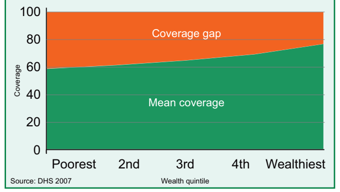

Source: DHS 2007

POLICIES

International Code of Marketing of Breastmilk Substitutes	Partial
New ORS formula and zinc for management of diarrhoea	Yes
Community treatment of pneumonia with antibiotics	Yes
IMCI adapted to cover newborns 0-1 week of age	Yes
Costed implementation plan(s) for maternal, newborn and child health available	Partial
Midwives be authorised to administer a core set of life saving interventions	Partial
Maternity protection in accordance with ILO Convention 183	No
Specific notification of maternal deaths	Partial

SYSTEMS

Financial Flows and Human Resources

Per capita total expenditure on health (US$)	42	(2007)
General government expenditure on health as % of total government expenditure (%)	8	(2007)
Out-of-pocket expenditure as % of total expenditure on health (%)	65	(2007)
Density of health workers (per 10,000 population)	5.8	(2005)
Official Development Assistance to child health per child (US$)	3	(2007)
Official Development Assistance to maternal and neonatal health per live birth (US$)	8	(2007)
National availability of Emergency Obstetric Care services (% of recommended minimum)	---	

Bangladesh
