# Supplementary material for: National health policy-makers’ views on the clarity and utility of Countdown to 2015 country profiles and reports: findings from two exploratory qualitative studies
Source: Health Res Policy Syst. 2014 Aug 15;12:40. doi: 10.1186/1478-4505-12-40 (PMC4139135; doi:10.1186/1478-4505-12-40)
Supplement: Additional file 3 — Countdown to 2015 country profile for Ghana, 2012. Source: Countdown to 2015: Maternal, Newborn & Child Survival. [file 1478-4505-12-40-S3.pdf]

Ghana

DEMOGRAPHICS

Total population (000)	24,392	(2010)
Total under-five population (000)	3,533	(2010)
Births (000)	770	(2010)
Birth registration (%)	71	(2008)
Total under-five deaths (000)	57	(2010)
Neonatal deaths: % of all under-5 deaths	38	(2010)
Neonatal mortality rate (per 1000 live births)	28	(2010)
Infant mortality rate (per 1000 live births)	50	(2010)
Stillbirth rate (per 1000 total births)	22	(2009)
Total maternal deaths	2,700	(2010)
Lifetime risk of maternal death (1 in N)	68	(2010)
Total fertility rate (per woman)	4.2	(2010)
Adolescent birth rate (per 1000 women)	70	(2006)

Under-five mortality rate

Deaths per 1,000 live births

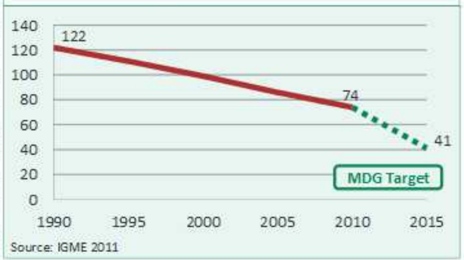

Source: IGME 2011

Maternal mortality ratio

Deaths per 100,000 live births

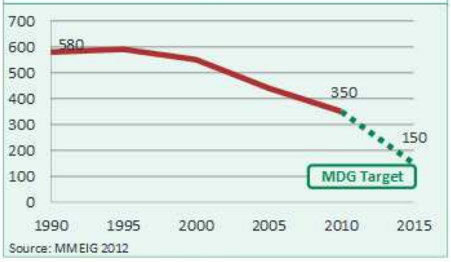

Source: MMEIG 2012

Note: MDG target calculated by Countdown to 2015

MATERNAL AND NEWBORN HEALTH

Coverage along the continuum of care

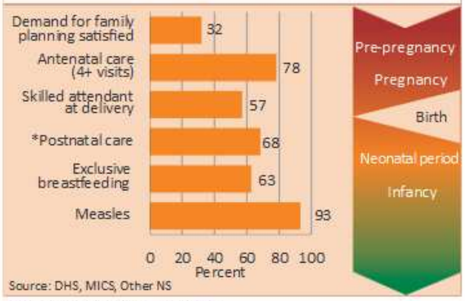

Source: DHS, MICS, Other NS

* See Annex/website for indicator definition

Skilled attendant at delivery

Percent live births attended by skilled health personnel

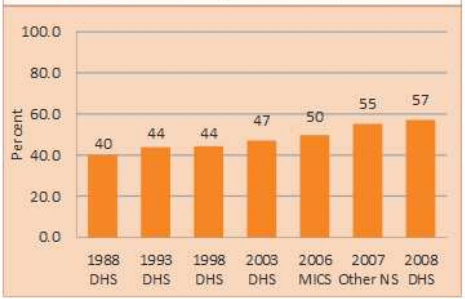

Prevention of mother-to-child transmission of HIV

Eligible HIV+ pregnant women receiving ART for their own health (%, of total ARVs)	0 (2010)

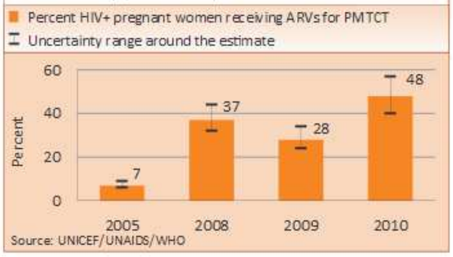

Source: UNICEF/UNAIDS/WHO

EQUITY

Socioeconomic inequities in coverage

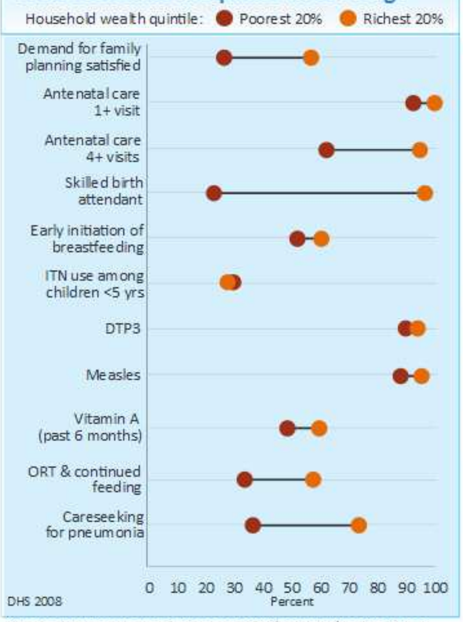

DHS 2008

Coverage levels are shown for the poorest 20% (red circles) and the richest 20% (orange circles). The longer the line between the two groups, the greater the inequality. These estimates may differ from other charts due to differences in data sources.

CHILD HEALTH

Immunization

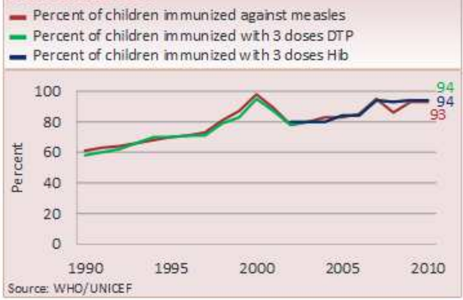

Source: WHO/UNICEF

Pneumonia treatment

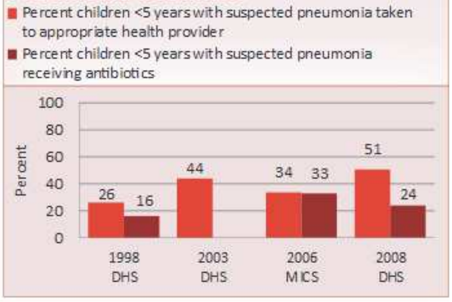

NUTRITION

Wasting prevalence (moderate and severe, %)	9	(2008)	Early initiation of breastfeeding (within 1 hr of birth, %)	52	(2008)
Low birthweight incidence (moderate and severe, %)	13	(2008)	Introduction of solid, semi-solid/soft foods (%)	76	(2008)
			Vitamin A supplementation (two dose coverage, %)	93	(2010)

Underweight and stunting prevalence

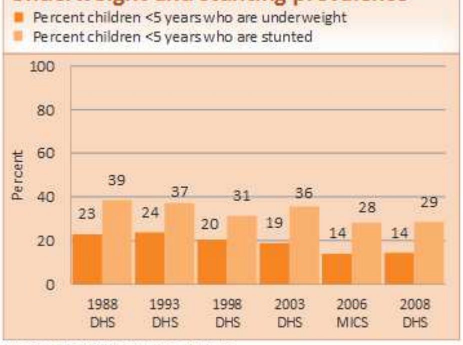

Note: Based on 2006 WHO reference population

Exclusive breastfeeding

Percent infants <6 months exclusively breastfed

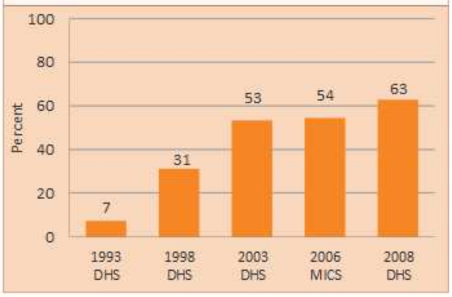

DEMOGRAPHICS

Causes of under-five deaths, 2010

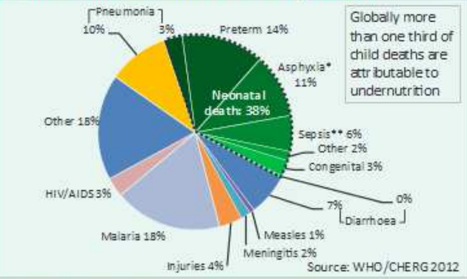

Source: WHO/CHERG 2012

*Intrapartum-related events **Sepsis/meningitis/tetanus

Causes of maternal deaths, 1997-2007

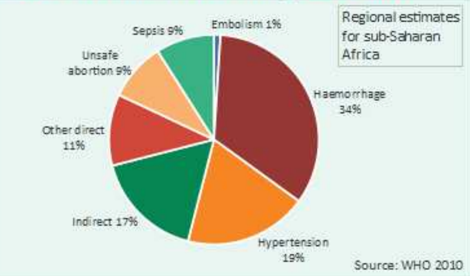

Source: WHO 2010

MATERNAL AND NEWBORN HEALTH

Antenatal care

Percent of women aged 15-49 years attended at least once by a skilled health provider during pregnancy

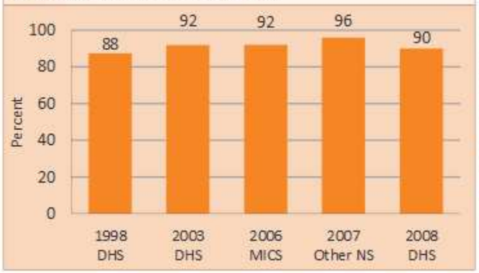

Demand for family planning satisfied (%)	32	(2008)
Antenatal care (4 or more visits, %)	78	(2008)
Malaria during pregnancy - intermittent preventive treatment (%)	44	(2008)
C-section rate (total, urban, rural; %) (Minimum target is 5% and maximum target is 15%)	7, 11, 5	(2008)
Neonatal tetanus vaccine (%)	86	(2010)
Postnatal visit for baby (within 2 days for all births, %)	-	-
Postnatal visit for mother (within 2 days for all births, %)	68	(2008)
Women with low body mass index (<18.5 kg/m², %)	8	(2008)

CHILD HEALTH

Diarrhoeal disease treatment

■ Percent of children <5 years with diarrhoea receiving oral rehydration therapy/increased fluids with continued feeding

■ Children <5 years with diarrhoea treated with ORS

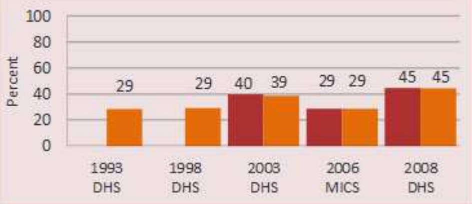

Malaria prevention and treatment

Percent of children receiving first line treatment among those receiving any antimalarial	50	(2008)

■ Percent of children <5 years sleeping under ITNs

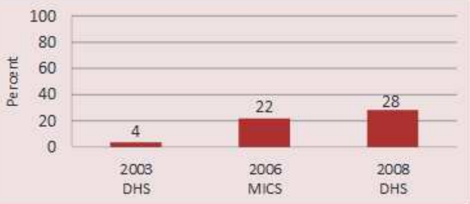

WATER AND SANITATION

Improved drinking water coverage

Percent of population by type of drinking water source, 1990-2010

■ Piped on premises ■ Other improved ■ Unimproved ■ Surface water

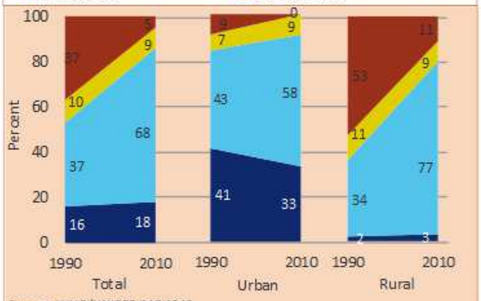

Source: WHO/UNICEF JMP 2012

Improved sanitation coverage

Percent of population by type of sanitation facility, 1990-2010

■ Improved facilities ■ Shared facilities ■ Unimproved facilities ■ Open defecation

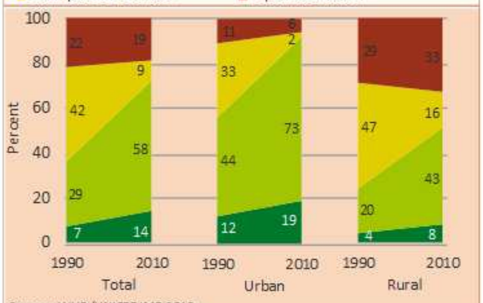

Source: WHO/UNICEF JMP 2012

POLICIES

Maternity protection in accordance with Convention 183	Partial
Specific notification of maternal deaths	Yes
Midwifery personnel authorized to administer core set of life saving interventions	Yes
International Code of Marketing of Breastmilk Substitutes	Yes
Postnatal home visits in first week of life	Yes
Community treatment of pneumonia with antibiotics	Yes
Low osmolarity ORS and zinc for management of diarrhoea	Yes
Rotavirus vaccine	Partial
Pneumococcal vaccine	Partial

SYSTEMS AND FINANCING

Costed national implementation plan(s) for maternal, newborn and child health available	Yes	
Density of doctors, nurses and midwives (per 10,000 population)	11.4	(2009)
National availability of emergency obstetric care services (% of recommended minimum)	37	(2011)
Per capita total expenditure on health (Int$)	325	(2010)
General government expenditure on health as % of total government expenditure (%)	12	(2010)
Out-of-pocket expenditure as % of total expenditure on health (%)	27	(2010)
Official development assistance to child health per child (US$)	21	(2009)
Official development assistance to maternal and neonatal health per live birth (US$)	43	(2009)

Countdown to 2015
Maternal, Newborn & Child Survival
